# Structural Insights into the Substrate Transport Mechanisms in GTR Transporters through Ensemble Docking

**DOI:** 10.3390/ijms23031595

**Published:** 2022-01-29

**Authors:** Carlos Peña-Varas, Christa Kanstrup, Ariela Vergara-Jaque, Mariela González-Avendaño, Christoph Crocoll, Osman Mirza, Ingo Dreyer, Hussam Nour-Eldin, David Ramírez

**Affiliations:** 1 Instituto de Ciencias Biomédicas, Universidad Autónoma de Chile, Llano Subercaseaux 2801-piso 6, Santiago 8900000, Chile; carlos.pena01@uautonoma.cl; 2 DynaMo Center, Department of Plant and Environmental Sciences, University of Copenhagen, 1871 Frederiksberg, Denmark; cka@plen.ku.dk (C.K.); chcr@plen.ku.dk (C.C.); huha@plen.ku.dk (H.N.-E.); 3 Center for Bioinformatics, Simulation and Modeling (CBSM), Faculty of Engineering, Campus Talca, Universidad de Talca, 1 Poniente No. 1141, Casilla 721, Talca 3460000, Chile; arvergara@utalca.cl (A.V.-J.); mariela.ag91@gmail.com (M.G.-A.); idreyer@utalca.cl (I.D.); 4 Department of Drug Design and Pharmacology, Faculty of Health and Medical Sciences, University of Copenhagen, 2200 Copenhagen, Denmark; om@sund.ku.dk; 5 Research Center for the Development of Novel Therapeutic Alternatives for Alcohol Use Disorders, Santiago 8900000, Chile

**Keywords:** glucosinolates, GTRs, ensemble docking, phytocompounds transport mechanism, membrane protein modeling

## Abstract

Glucosinolate transporters (GTRs) are part of the nitrate/peptide transporter (NPF) family, members of which also transport specialized secondary metabolites as substrates. Glucosinolates are defense compounds derived from amino acids. We selected 4-methylthiobutyl (4MTB) and indol-3-ylmethyl (I3M) glucosinolates to study how GTR1 from *Arabidopsis thaliana* transports these substrates in computational simulation approaches. The designed pipeline reported here includes massive docking of 4MTB and I3M in an ensemble of GTR1 conformations (in both inward and outward conformations) extracted from molecular dynamics simulations, followed by clustered and substrate–protein interactions profiling. The identified key residues were mutated, and their role in substrate transport was tested. We were able to identify key residues that integrate a major binding site of these substrates, which is critical for transport activity. In silico approaches employed here represent a breakthrough in the plant transportomics field, as the identification of key residues usually takes a long time if performed from a purely wet-lab experimental perspective. The inclusion of structural bioinformatics in the analyses of plant transporters significantly speeds up the knowledge-gaining process and optimizes valuable time and resources.

## 1. Introduction

The nitrate/peptide transporter family (NPF) is one of the largest transporter families in the plant kingdom. It belongs to the major facilitator superfamily (MFS) of membrane proteins and has arisen as an essential transporter family of many substrates, including ions such as nitrate and chloride, as well as phytochemical compounds such as peptides, amino acids, glucosinolate defense compounds, and plant hormones such as auxin, abscisic acid, giberellins, and jasmonates [1,2].

The number of crystal structures available for members of the NPF family is low. For instance, currently in the PDB database there are only three crystal structures deposited that belong to the NPF, all of which belong to the *Arabidopsis thaliana* NTR1.1 (NPF6.3) transporter (PDB codes 4OH3 [3], 5A2O, and 5A2N [2]). This transporter has been reported to be a dual-affinity nitrate transporter and the crystallization studies have mainly focused on elucidating the structural basis for nitrate transport. The NPF belongs to the proton-dependent oligopeptide transport family which is found in all kingdoms. In comparison to plants, the POTs are typically represented by much fewer members per organism. For example, Arabidopsis contains 53 NPFs, whereas humans only contain four POTs. The pharmacological relevance of the human Pet1 and PepT2 (involved in the uptake of peptidomimic drugs [4]) has driven research into understanding how POTs transport oligopeptides. Most POT crystals came from bacterial homologs such as YbgH [5], GkPOT [6], PepT_St_ [7], PepT_So_ [7], and PepT_So2_ [8,9].

Based on these studies, a transport model is proposed wherein the POT transporters recognize and bind their substrate via a discrete set of amino acid residues on one side of the cell membrane, then alternate between at least two conformations, the outward (ground state of an electrogenic transporter) and the inward (excited state) conformation, to expose the binding site to the opposite side of the bilayer [10]. During its path through a transporter, the substrate interacts with an additional set of amino acid residues until it is released. These sets of key amino acid residues are known as “molecular determinants”. Transition from outward to inward state is energized by protonation at the pore, while the conformational change from excited to ground state is spontaneous [11]. A fundamental feature for substrate transport by POT transporters is the highly conserved motif EXXEX [12,13]. This motif has a central role in H^+^ coupling [5,6,7,14,15], and has also been found to be essential for the active transport of the 4MTB (4-methylsulfinylbutyl) glucosinolate by GTR2, as the mutations E57A, E60A, and K61A caused a substantial reduction of 4MTB accumulation [15]. Moreover, it has been reported that by mutating the charged amino acids, the NO_3_^−^ uptake activity is eliminated in the NTR1.1 transporter [3]. Besides these insights, we know very little about the molecular determinants of substrate specificity towards organic substrates in the NPF.

Glucosinolates are defense compounds derived from amino acids [16]; structurally, they are anions composed of thiohydroximates linked with three moieties: a sulfate, β-glucopyranosyl, and an variable side chain (amino acid-derived) [17]. We selected these phytochemical compounds as a model to study the specificity and the transport mechanism in the NPF family. Biophysical characterization using two-electrode voltage clamp (TEVC) showed that GTR1 and GTR2 were able to transport different methionine-derived glucosinolates such as 4MTB, 4-methylsulfinylbutyl-(4MSB), 3-methylsulfinylpropyl-(3MSP), and p-hydroxybenzyl-(pOHB) glucosinolates, as well as NO_3_^−^ [18] and tryptophan-derived glucosinolates. In contrast, GTR3, which shares 60% of sequence identity with GTR1, strongly prefers tryptophan-derived glucosinolates such as indol-3-ylmethyl (I3M) glucosinolate [19].

In the work presented here, we have designed a systematic computational protocol for the rapid identification of potential molecular determinants involved in plant substrate translocation across the membrane, using glucosinolates as model system to study transport mechanism in the NPF family. We implemented and automated a pipeline that includes massive docking of the substrates 4MTB and I3M in an ensemble of GTR1 conformations extracted from molecular dynamics simulations, followed by clustering and substrate–protein interactions profiling. In this way, we were able to identify potential key residues that integrate a major binding site of these substrates, which is critical for transport activity.

## 2. Results

### 2.1. Molecular Modeling of GTR1 in Both Inward- and Outward-Facing Conformations

To simulate the behavior of GTR1 in both inward- and outward-facing conformations, different models were constructed (Table 1). First, we modeled the missing amino acids in the NTR1.1_in_ crystallographic structure (PDB code: 4OH3), then GTR1_in_ was modeled using NTR1.1_in_ as template. Sequence identity between GTR1 and NTR1.1 was 30.3% (Figure S1). In the GTR1_in_ final model, ≈99% of the residues showed a high stereochemical quality, as evaluated by PROCHECK. The global ProQM score obtained for GTR1_in_ was 0.637, where 0.7 is typical for membrane protein structures solved by X-ray crystallography.

**Figure 1:**
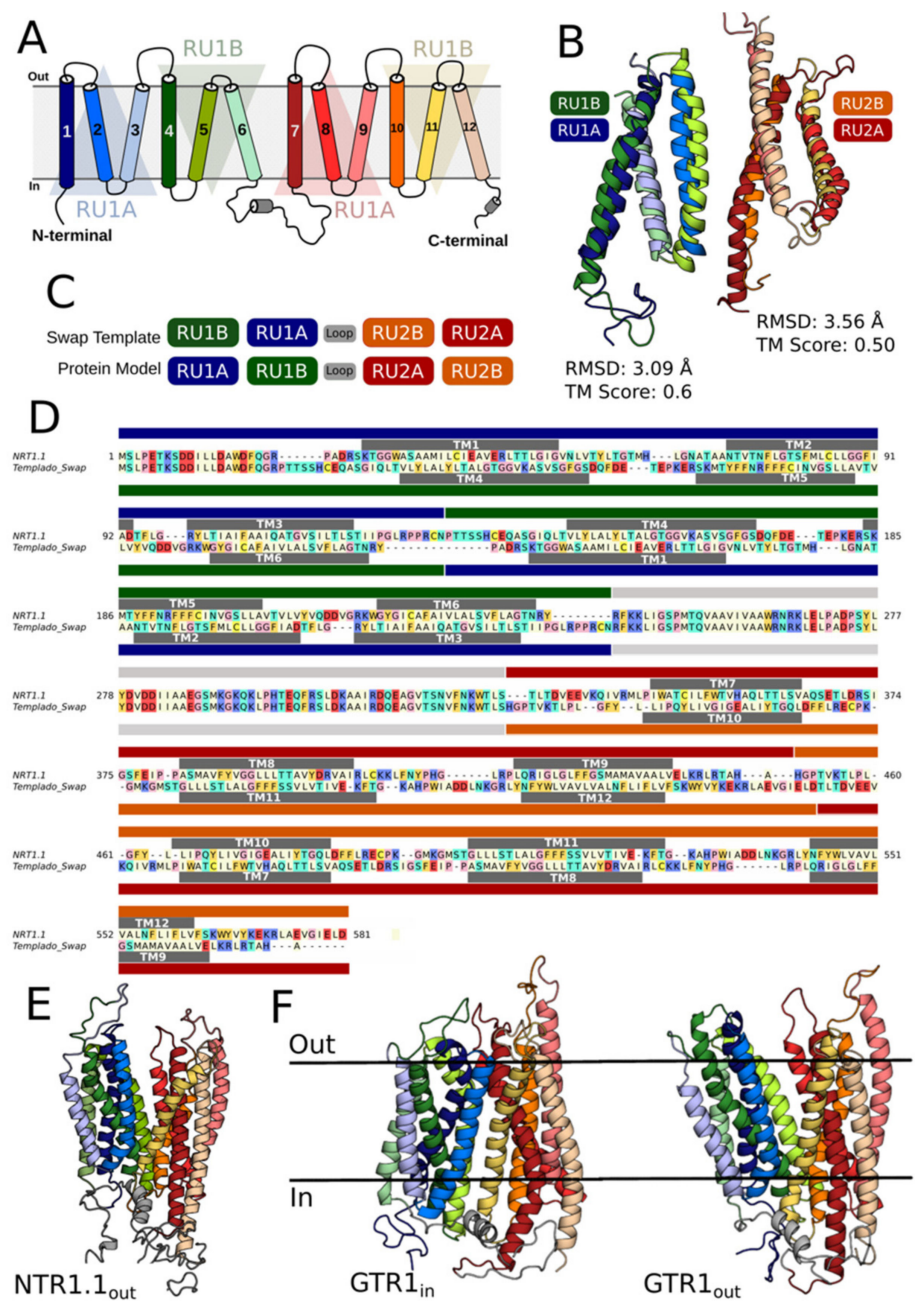
Modeling of NTR1.1_out_ and GTR1_in/out_. (**A**) Schematic representation of NTR1.1 topology with the N- and C-terminus exposed to the intracellular medium. The helices on the blue and green triangular backgrounds comprise repeat unit 1 A (RU1A) and 1 B (RU1B), respectively, while repeat unit 2 A (RU2A) and 2 B (RU2B) are composed of the helices on the red and orange triangular backgrounds. (**B**) RU1A and RU2A are related to RU1B and RU2B by two-fold pseudosymmetry. A structural alignment, built with TM-Align, of the repeats is shown in cartoon representation with the helices colored according to the topology. The RMSD and TM score resulting from the structural superimposition of RU1A/RU1B and RU2A/RU2B in GTR1 are given in each case. (**C**) Schematic representation of the sequence alignment used to build the NTR1.1_out_ swapped-repeat model. (**D**) Refined sequence alignment between the NTR1.1 model and swapped-template sequences. The alignment is colored according to the chemical properties of the residues. The secondary structure (helix) assignment is indicated by dark grey rectangles and labeled by TM segments. The sequence range of each repeat is displayed with bars above and under the target and template sequences, respectively, colored according to the triangular backgrounds in (**A**). (**E**) Final NTR1.1 model in an outward-facing conformation. (**F**) Final models of GTR1_in_ and GTR1_out_.

To model GTR1 in an outward-facing conformation, we first modeled the NTR1.1_out_ conformation employing the repeat-swap homology modeling technique (see Materials and Methods section). Then, GTR1_out_ was modeled using the NTR1.1_out_ conformation as template. The final GTR1_out_ model showed ≈98% of the residues in favored regions of the Ramachandran plot, while the ProQM score was 0.646. Figure 1 illustrates the modeling process of NTR1.1_out_ and GTR1 in both inward- and outward-facing conformations.

**Scheme 1:**
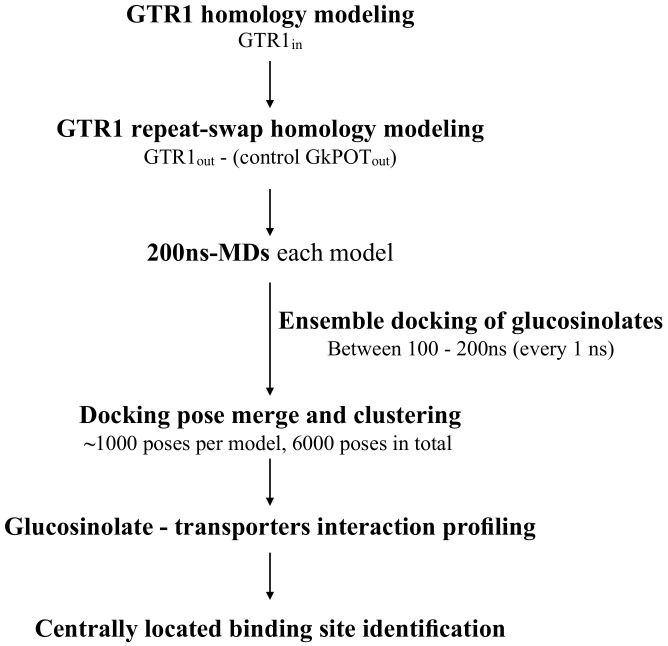
Flow chart summarizing the steps followed in the present work to formulate a hypothesis about the configuration of glucosinolates 4MTB and I3M binding site in glucosinolate transporter GTR1.

As positive control to test the accuracy of our docking pipeline (see Scheme 1 in Materials and Methods section), the crystal structure of the peptide transporter GkPOT in complex with alafosfalin (PDB code: 4IKZ) [6] was used. As the crystallographic structure was resolved in an inward-facing conformation, an outward-facing structural model of the refined GkPOT structure was also built through the repeat-swap homology modeling technique (GkPOT_out_). The best repeat-swapped GkPOT_out_ model showed ≈99.2% of the residues in favored regions of the Ramachandran plot, while the ProQM score reached a value of 0.650.

### 2.2. Generation of GTR1 Ensemble Conformations

The GkPOT_in_ crystallographic structure and GTR1_in_ and GTR1_out_ homology models, as well as the GkPOT_out_ repeat-swapped model, were subjected to 200 ns–MDs and then we extracted an ensemble of conformations for each structure, featuring the largest structural variance of the amino acids that are supposed to build the substrate pathway. The RMSDs of the position for all protein atoms of both GTR1 models from their initial configuration as a function of simulation time are illustrated in Figure S3. We test the behavior of GTR1 models during unrestricted MDs, but we noticed that some alpha helices lose their secondary structure as the simulation progresses. We speculate that this is because the models (mainly those in outward conformation) were not fully stable. For this reason, a restraint spring constant to 1 kcal × mol^−1^ × Å^−2^ was applied to the secondary structure of each transporter during the 200 ns of production simulation to keep its secondary structure stable, and to give flexibility to the amino acid side chains.

All transporters were equilibrated after 30 ns of MDs (Figure S3). The RMSD values remain within 0.6 Å for all models, demonstrating the conformational stabilities of the receptor structures. For each model, 100 structures were extracted and aligned from the last 100 ns of simulation. We obtained, in total, 400 structures: 100 for each model (GTR1_in_, GTR1_out_, GkPOT_in_, and GkPOT_out_).

### 2.3. Testing the Ensemble Docking Pipeline

**Figure 2:**
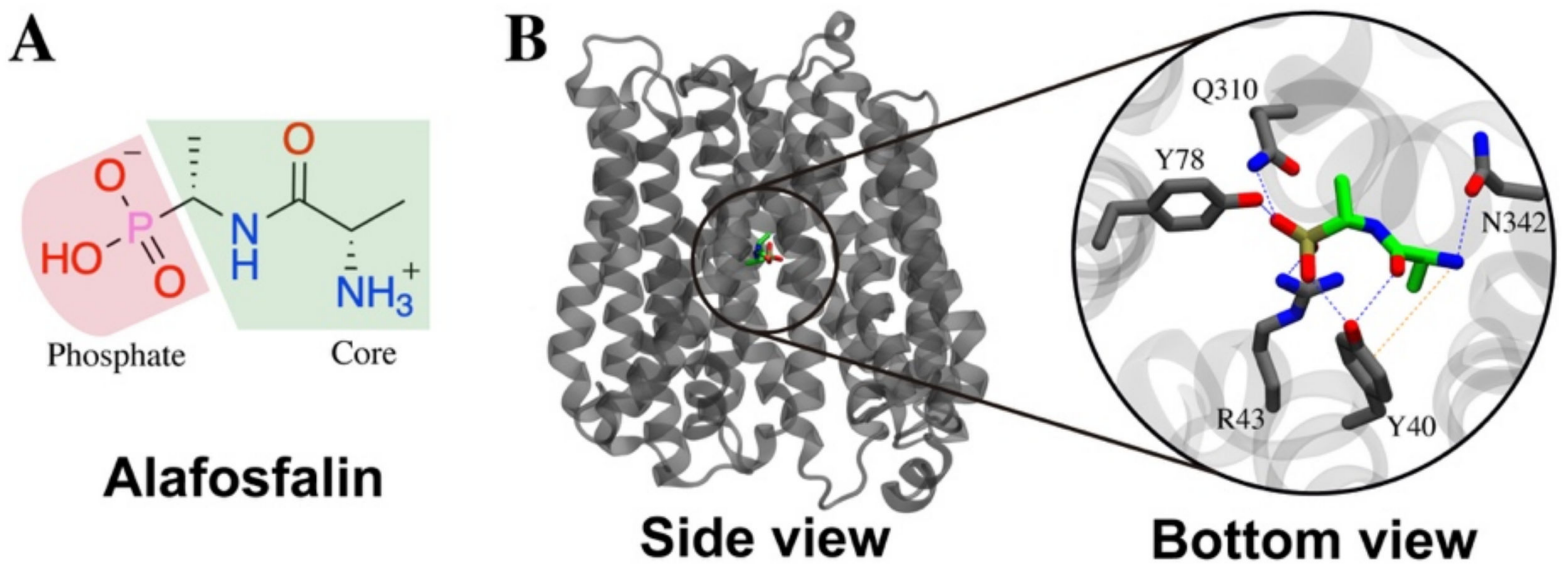
Alafosfalin binding site into GkPOT_in_. (**A**) Chemical structure of alafosfalin. (**B**) Alafosfalin binding site into GkPOT in an inward-facing conformation (GkPOT_in_). Hydrogen bond and Pi–cation interactions are displayed as blue and orange dotted lines, respectively.

To test if massive docking applied to an ensemble of conformations derived from MDs (Scheme 1) is a useful tool to identify the key residues of a transporter that interact with a substrate at the central binding site, we used GkPOT in complex with alafosfalin as a positive control (PDB code: 4IKZ). First, we took the GkPOT–alafosfalin complex and generated the interaction profile using the *poseviewer_interactions.py* script (see Materials and Methods). Hereby, we split alafosfalin in two moieties, “phosphate” and “core” (Figure 2A). We found that the alafosfalin phosphate interacted with residues Y40, R43, Y78, and Q310 through several hydrogen bonds, while the alafosfalin core also established hydrogen bonds with Y40 and N342, as well as a cation–Pi interaction with Y40 (Figure 2B). These results were in line with those reported by Doki et al. [6] and allowed us to trust the script used for characterizing the interactions of the substrates with their respective targets.

**Figure 3:**
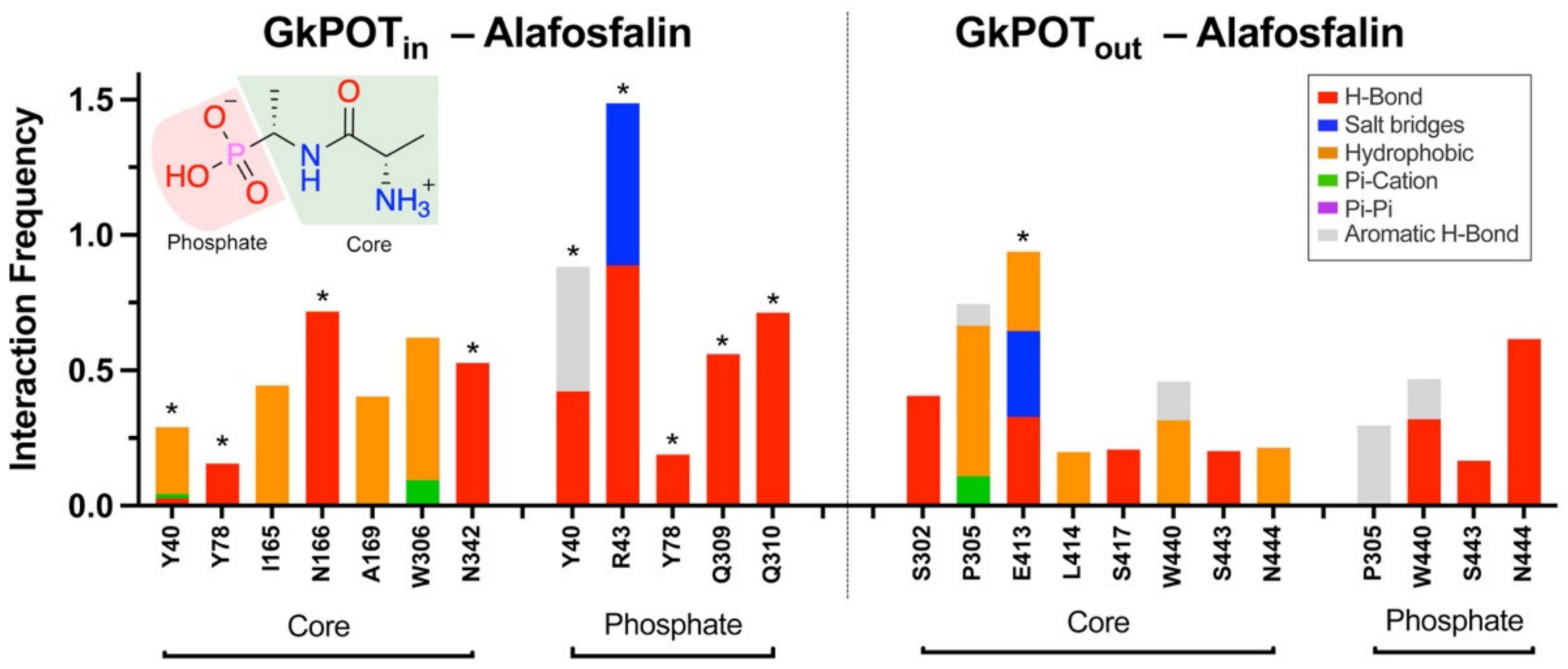
Contacts of alafosfalin with residues of the GkPOT in an inward (GkPOT_in_) and outward (GkPOT_out_) conformation, and the nature of the chemical interactions. Interactions between the GkPOT residues and alafosfalin are categorized into six types: Hydrogen bonds (H-bond), salt bridges, hydrophobic, Pi–cation, Pi–Pi, and aromatic hydrogen bonds. The stacked bar charts are normalized over the interactions between significant clusters conformers and GkPOT structures. The value could be greater than 1 because the same residue might establish more than one interaction with the ligand. Interactions with a frequency greater than 0.15 are shown. Reported interactions are denoted with *.

Next, we docked alafosfalin into an ensemble of GkPOT_in_/GkPOT_out_ conformations and found four significant clusters for GkPOT_in_ as well as four for GkPOT_out_ (Table 2). The interaction profile of alafosfalin in complex with GkPOT in both conformations was determined (Figure 3) and we found that interactions described in the crystallographic structure (Figure 2B) were conserved. In addition, we found other contacts that could be important for alafosfalin interactions with a centrally located binding site in GkPOT. Due to the nature of the substrate, the main interactions were established as hydrogen bonds and hydrophobic contacts. With our ensemble docking pipeline, we could identify the key residues Y40, R43, Y78, and Q310, described in the literature as residues involved in peptide specificity, N166 and N342 involved in peptide binding [6], Q309 involved in peptide binding/protonation [2,3], and E413 involved in proton translocation [6]. Most of the reported key interactions were identified in the GkPOT_in_ conformation, which was reasonable because this conformation was directly based on the crystal structure of the transporter.

**Figure 4:**
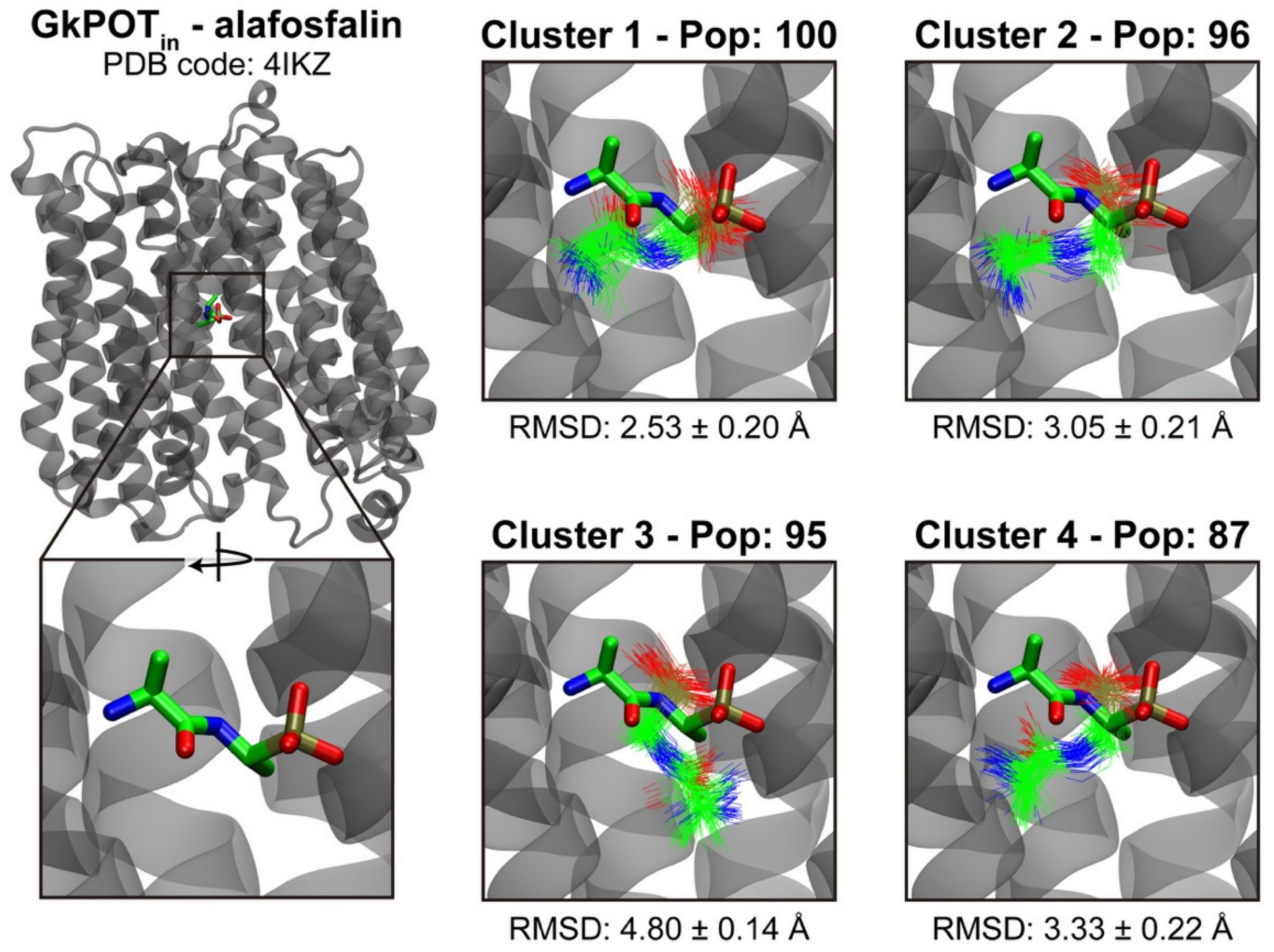
Ensemble docking of alafosfalin into GkPOT. Crystal structure of GkPOT_in_ in complex with alafosfalin (left). Significant clusters of alafosfalin conformers from ensemble docking (line representation) with respect to reference ligand from the crystal structure 4IKZ (stick representation).

As it was only possible to compare alafosfalin docking results with GkPOT_in_, because the crystallographic structure is in an inward conformation (PDB code: 4IKZ), we calculated the root mean square deviation (RMSD) of each pose in all significant clusters against its conformation in the crystal structure. The most populated cluster (cluster 1—Table 2) was the one with the lowest RMSD (2.53 ± 0.2 Å), indicating that with our docking ensemble pipeline it was indeed possible to reproduce the binding mode of a substrate to a transporter (alafosfalin to GkPOT_in_). We also found that the phosphate moiety of all conformers was always in the same orientation (Figure 4), anchored to the binding site through several H-bond interactions with Y40, R43, Y78, Q309, and Q310 (Figure 3), whereas the alafosfalin core was more flexible and moved around the cavity. Our computational results were in good correlation with the experimental results reported for this substrate [6].

### 2.4. Glucosinolate Centrally Located Binding Site Revealed by Ensemble Docking

After having validated the method, we then employed it to investigate the interaction between glucosinolates and GTR1. The aliphatic and indole glucosinolates 4MTB and I3M were docked into an ensemble of conformations of GTR1 in both inward and outward configurations. For each configuration, a total of 100 conformations were extracted from molecular dynamics simulations and then aligned against the original model. For each docking run (four runs in total, two substrates, GTR1 in two configurations—Table 3), the top 10 poses were selected and merged, resulting in ~1000 substrate poses on each GTR1 conformation. Then, all conformers were clustered using an RMSD matrix (see Materials and Methods section). Several clusters per system were obtained (Table S1), with populations between 1 to 510 conformers. Significant conformational clusters, for which the populations departed by more than twice the standard deviation from the average cluster population, are summarized in Table 3. We observed that 4MTB conformers of the significant clusters in GTR1_in_ and GTR1_out_ constitute around the 50% of the total number of poses obtained in the ensemble docking in these systems, whereas I3M conformers integrated around 37% of the total conformations. This indicated that, indeed, the most visited poses by the substrates were those that surely represent the best way in which interactions were established with the main binding site of the glucosinolate transporters. Figure S5 displays all significant clusters. Although a grid box was chosen that was large enough to allow interactions along the entire substrate translocation pathway (Figure S4), the significant interactions of both 4MTB and I3M glucosinolates with GTR1 focused always on a specific zone. These results further increased our confidence in the method, because when the same docking ensemble process was employed for GkPOT and alafosfalin, the significant interaction poses of this substrate always focused on the same site where the reference ligand was cocrystallized (Figure 4), indicating that there was a centrally located binding site. This biding site was characterized by the highest energetic stability and showed the most stable interactions with the substrate. The fact that this binding site could be identified using ensemble docking simulations strongly supports the validity of the chosen method.

### 2.5. Contacts of Glucosinolates with Key Residues of the Centrally Located Binding Site and the Nature of the Chemical Interactions

**Figure 5:**
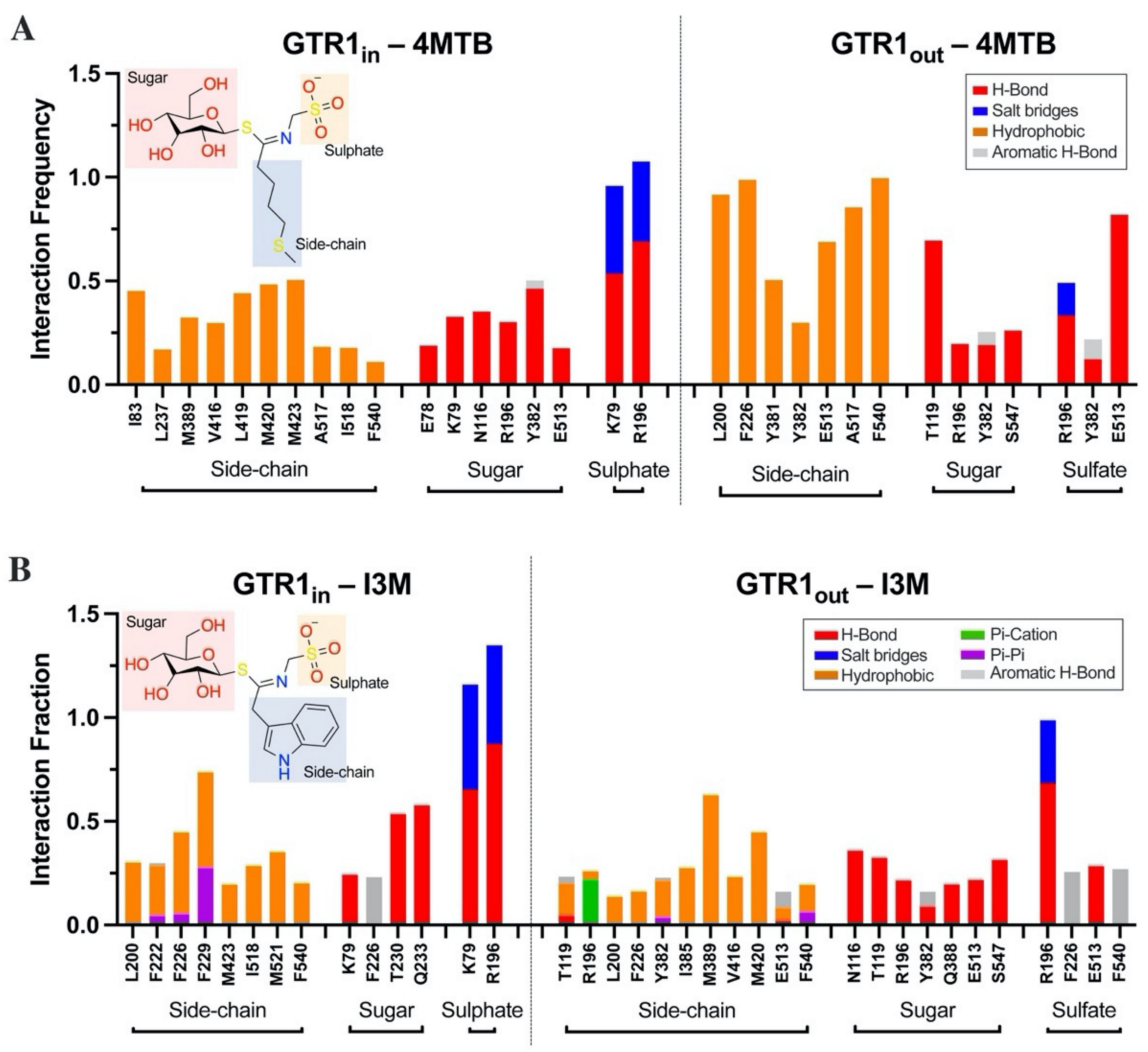
Contacts of 4MTB (**A**) and I3M (**B**) with residues of the GTR1 in an inward (GTR1_in_) and outward (GTR1_out_) conformation, and the nature of the chemical interactions. Interactions between the GTR1 residues and glucosinolates are categorized into six types: Hydrogen bonds (H-bond), salt bridges, hydrophobic, Pi–cation, Pi–Pi, and aromatic hydrogen bonds. The stacked bar charts are normalized over the interactions between significant clusters conformers and GTR1 structures. The value could be greater than 1 as the same residue might establish more than one interaction with the ligand. Interactions with a frequency greater than 0.15 are shown.

To profile the interactions of both glucosinolates (4MTB and I3M), we broke down the ligands into three different moieties: sugar and sulfate, which comprised the common backbone of the substrates, as well as the side-chain, which represented the sole chemical difference between the two glucosinolates (Figure 5). In this way we were able to observe not only how the whole ligand interacted with GTR1 (classical molecular docking analysis approach), but also what portion of the ligand established the key interactions. This type of more thorough analysis allowed us to fine-tune which segments of the ligand were relevant according to the type of interaction being established. In this work we fully automated the analysis using a KNIME workflow. For example, in the case of glucosinolates, we could observe that highly conserved H-bond and ionic (salt bridges) interactions were established between the sulfate of both glucosinolates and residues K79 when the substrates interacted with GTR1_in_. K79 has been characterized as a key amino acid in the ExxE[K/R] motif that is essential for proton-coupling and active glucosinolate [15] and peptide [6] transport (Table 4). Key interactions with R196 were also identified for both glucosinolates when interacting with GTR1 in both conformations (Figure 5).

Different hydrogen bond interactions between the sugar of both glucosinolates and GTR1 were also well characterized, including residues K79 and R196, as well as N116, Y382, E513, and S547. These H-bond interactions were crucial to stabilize the substrates in the centrally located binding site. In addition, common interactions were found in both inward and outward conformations (sugar with N116, Y382, E513), whereas interactions with S547 were only found when the substrates interact with GTR1_out_. We concluded that certain residues were involved in the transition between states (inward-to-outward and outward-to-inward), and that others were more specialized and only interacted with the substrate in a fixed conformation due to their location in the binding site. Furthermore, as expected, the side-chain interactions of both glucosinolates were mostly hydrophobic. Additionally, I3M also presented Pi–Pi and Pi–cation interactions due to the aromatic nature of this radical (Figure 5B). Common hydrophobic contacts were found for both substrates with residues L200, F226, Y382, M389, V416, M423, I518, and F540 (Figure 5).

**Figure 6:**
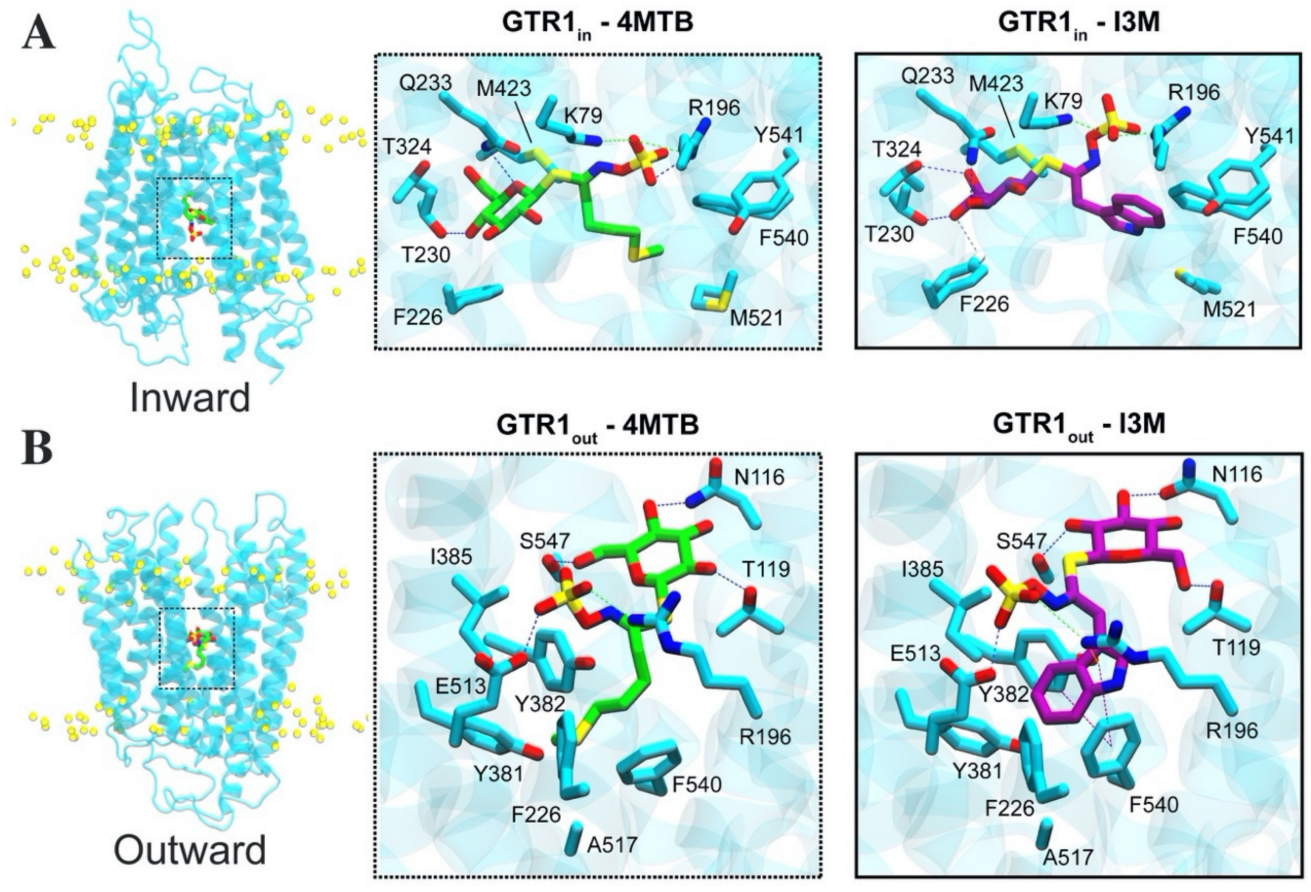
Contacts of 4MTB and I3M with GTR1 in both inward (**A**) and outward (**B**) conformation. Hydrogen bonds (blue), salt bridges (green), Pi–Pi (purple), Pi–cation (orange), and aromatic hydrogen bond (gray) interactions are displayed as colored dotted lines. GTR1_in_-4MTB, GTR1_out_-4MTB, GTR1_in_-I3M, and GTR1_out_-I3M are conformers from clusters with population = 53, 510, 186, and 88, respectively (Table 3). Polar heads of membrane bilayer are displayed as yellow spheres (left panels); phospholipids tails are not displayed to improve quality of the figures.

When glucosinolate conformers from significant clusters (see Section 4.4) are analyzed, it is evident that 4MTB and I3M interact with GTR1 in a similar way. It is observed that the side chain is located pointing downwards when both substrates interact with the two GTR1 conformations studied. It is also observed that sulfate is anchored by different interactions, where those established with K79 and R196 in the GTR1_in_ conformation, as well as R196 and E513 in the GTR1_out_ conformation, are highlighted. Common sugar interactions are also observed, as well as mainly hydrophobic interactions of the side chain (Figure 6).

Table 4 summarizes the residues identified in this study by ensemble docking and those reported to be essential for glucosinolate and/or peptide transport. It is observed that several key residues in the ExxE[K/R] motif were identified in this study, including E78, K79, and R196. Additionally, homologous residues in POTs that are responsible for substrate binding and specificity were identified, including I83, N116, T230, and L419. Finally, residue E513, which is responsible for proton translocation in POTs, was also detected with our ensemble docking pipeline.

### 2.6. Glucosinolate Transport Is Governed by Ionic Interactions

**Figure 7:**
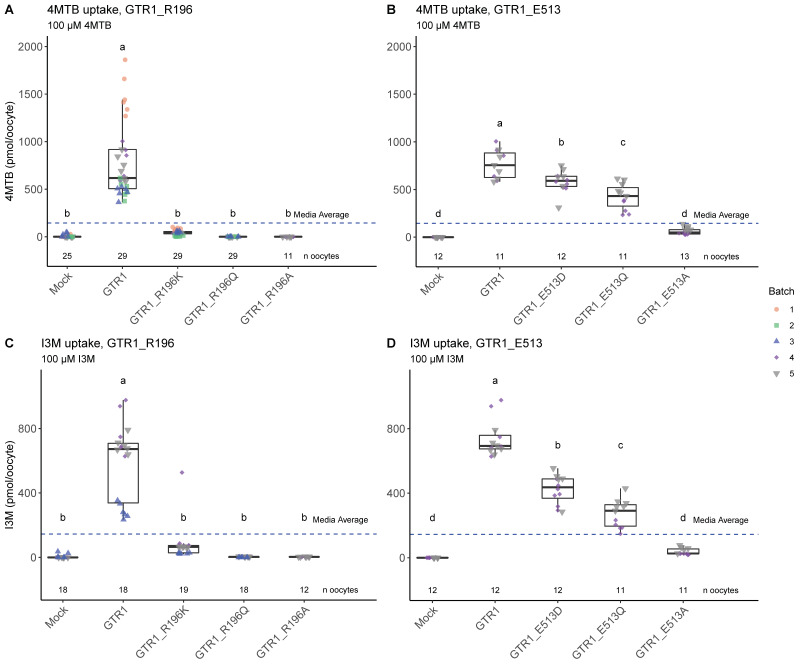
Oocytes exposed to 100 μM of either 4MTB or I3M in MES-based kulori pH 5 for 1 h. (**A**,**B**): uptake of 4MTB, (**C**,**D**): uptake of I3M. (**A**,**C**) display the uptake of the glucosinolates upon mutation of GTR1_R196. (**B**,**D**) display the uptake of the glucosinolates upon mutation of GTR1_E513. Uptake of each mutant version measured in at least two different batches of oocytes, with a minimum of five oocytes per batch. Points in different color and shape represent the different batches. Numbers in the bottom display *n* number of oocytes for each mutation (11–29 oocytes). The dotted blue line represents the average media level over all the assays. Statistical assessments were performed using a one-way ANOVA using Tukey’s HSD post hoc test, *p* < 0.05.

To corroborate whether the residues identified in this study are key in GTR1-mediated transport of 4MTB and I3M glucosinolates, residues R196 and E513 were mutated by R196K, R196Q, and R196A, as well as E513D, E513Q, and E513A. Then, we characterized their ability to actively transport both substrates (Figure 7). In the case of the R196 mutants, it was found that the slightest change of this residue drastically alters the ability of GTR1 to transport both substrates, as both R196K and R196A mutants presented the same results. Thus, apparently the interaction of this residue with the glucosinolate sulfate moiety is key for the active transport of this substrates. In the case of the E513 mutants, it is observed that when the change is made for another acidic residue (E513D), the transport of both glucosinolates is diminished, as well as with more drastic changes (E513Q). However, residue E513 appears to be relevant, as when the acidic side chain is removed (E513A), the active transport activity is eliminated, thus proving that this residue is also key in the binding and transport of both substrates (Figure 7). These findings corroborate that ionic interactions with R196 and E513 are essential for active transport of both aliphatic and aromatic glucosinolates.

## 3. Discussion

Glucosinolate transporter GTR1 is able to actively transport 4MTB and I3M, two glucosinolates that have different physicochemical characteristics (Figure 5), as one is derived from methionine (4MTB) and the other from tryptophan (I3M). To date, the key amino acids in the transport process of these substrates are not well known, and the approaches that have been followed to identify potential molecular determinants have been focused on in multiple sequence alignments following extensive validation [14,15]. In this work, we tackle this problem focusing on a structural bioinformatics approach, as we modeled GTR1 in two conformations (inward and outward) and generated an ensemble of conformers to add flexibility to the transporter. Subsequently, we performed a conformational sampling by massive molecular docking of both glucosinolates to study their interaction with the transporter. Ensemble docking of multiple protein structures has been successfully used to incorporate protein flexibility in molecular docking and thus study in a more detailed way how a ligand interacts with its target protein [22,23,24]. This methodology has also been applied to homology models [25] and difficult protein targets [26], as well as different transporters, such as the drug/proton antiporter AcrB [27,28] and serotonin transporters SERT [29]. It has never, before our study, been applied to the identification of key residues in plant transporters (in different inward/outward configurations) such as GTRs, nor has an analysis tool been provided to identify the key amino acids from all the ensemble docking solutions obtained.

To test whether by molecular docking it is possible to identify key amino acids for the transport of phytocompounds, the *Geobacillus kaustophilus* peptide transporter (GkPOT) in complex with alafosfalin was used as positive control (PDB code: 4IKZ). As a result, we observed that a broad conformational sampling, such as the one obtained by ensemble docking, allows identification of the important residues in substrate transport. In our case, we were able to identify (Figure 3 and Figure 4) the residues reported as essential in the transport of alafosfalin by GkPOT (Table 4) [6], and reproduce with our pipeline alafosfalin conformation in the crystal structure (Figure 4). Additionally, other residues not identified by crystallography in GkPOT were identified with our protocol, mainly hydrophobic interactions (I165, A169, W306, P305, and W440) and a hydrogen bond with N444 (Figure 3). In the future, it is important to design experiments to corroborate whether these interactions are indeed relevant for the transport of alafosfalin by GkPOT.

The main binding site of both glucosinolates was found in the central part of the pathway cavity (Figure S5). In both conformations, this centrally located binding site is in the most closed section. Therefore, when GTR1 alters between the outward (ground state of an electrogenic transporter) and the inward (excited state) conformations, the ligand does not move significantly, which allows the process to be energetically stable. This hypothesis is validated because when 4MTB and I3M were docked in GTR1_in_ and GTR1_out_, common interacting residues were found in the binding sites of these opposite conformations of GTR1. Between the most relevant interactions found for both substrates are hydrophobic contacts between the sidechain and residues L200, F226, and F540; hydrogen bonds between the sugar/sulfate moieties and GTR1_in_-K79 residue, as well as GTR1_out_ T119, R196, Y382, and S547 residues. We also identified key interactions between both glucosinolates and R196 when interacting with GTR1 in both conformations (inward and outward). The key residue R196 in peptide transporters (POT), homologous residue K136 in GkPOT. and K126 in PepT_St_, has been reported as a key ExxE[K/R]-interactor amino acid [6,19]; our analyses indicate that it interacts directly with glucosinolates. This leads us to hypothesize that this residue not only plays a key role in proton translocation when substrates are transported (this was not identified in the present work) but is also a molecular determinant in the translocation of different glucosinolates (aliphatic, aromatic, etc.) due to its conserved interaction with the sulfate at the backbone of these defense phytocompounds in both GTR1_in_ and GTR1_out_ conformations (Figure 6). Finally, it is possible to observe that, despite the clear differences between glucosinolates transported by GTR1 and peptides transported by POT (Figure 3 and Figure 5), the transport mechanisms have clear similarities as several of the residues identified in this study are homologous to key residues for binding specificity and transport of peptides (Table 4). This allows us to conclude that our pipeline (Scheme 1) is a valuable tool to rapidly identify amino acids with potential key roles not only in the transport of compounds across the cell membrane, but also in specificity.

**Figure 8:**
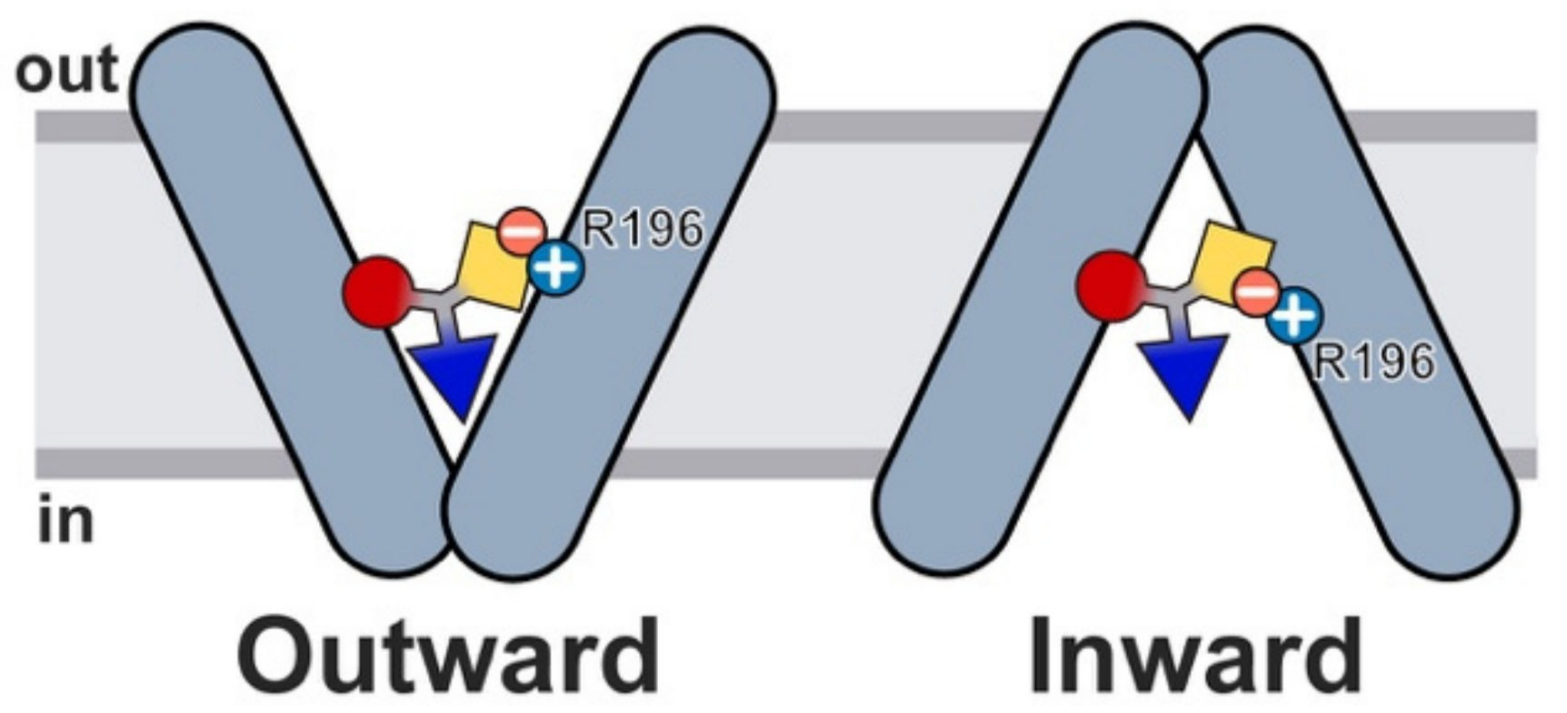
Proposed role of R196 in the glucosinolate structural transition between the outward and inward states. Glucosinolate is represented according to the moieties studied in this work: Sugar (red), sulfate (yellow), and sidechain (blue) (see Figure 5 for more details).

A biochemical analysis was performed by mutating residues R196 and E513 to amino acids of similar characteristics (R196K and E513D) and eliminating the basic or acidic character of both residues (R196A and E513A) (Figure 7). Combining the present ensemble docking results and the biochemical and biophysical analyses, we proposed R196 as key residue involved in the transport mechanism of glucosinolates (Figure 8). In this mechanism, GTR1_out_-R196 anchors the glucosinolate into the centrally located binding site through ionic contacts, then holds the substrate at the binding site during the outward-to-inward transition, and subsequently presents it in the inward-facing conformation so that the substrate is released into the intracellular space. Additionally, according to the different docking poses identified in the significant clusters, it appears that the most favorable orientation for the transition is where the substrate side chain is oriented towards the interior of the cell.

## 4. Materials and Methods

Scheme 1 summarizes the major steps followed in the present work to formulate a hypothesis about the configuration of glucosinolates 4MTB and I3M binding site in GTR1, which will be presented in more detail within the Materials and Methods section.

### 4.1. Molecular Modeling of GTR1

To model *Arabidopsis thaliana* GTR1 transporter in an inward-facing conformation (GTR1_in_), a template structure was identified using Psi-Blast v2.7.1 [30] on the nonredundant Protein Data Bank (PDB) database (). The crystal structure of the plant dual-affinity nitrate transporter NRT1.1 from *Arabidopsis thaliana* (PDB code: 4OH3) [3], in an inward-facing conformation, was selected as the most suitable template for modeling. The NRT1.1 gene encodes a 590-amino-acid protein; however, residues located in the terminal domains and external loops are missed from the crystal structure (i.e., residues 1–8, 282–295, 311–324, 451–458 and 582–590). To model these regions, the robotics-inspired kinematic closure (KIC) algorithm [31] implemented in the ROSETTA v3.8 software (Seattle, WA, USA) [32] was used. A total of 6000 conformations were generated to develop models of the small contiguous loops containing between 8 to 14 residues. The best NRT1.1 refined conformation was selected as that with the lowest ROSETTA score.

Once the template structure was refined, a pairwise sequence alignment between NTR1.1 and GTR1 was constructed using the AlignMe server v1.1 (Frankfurt am Main, Germany) in PS mode [33]. Then, a total of 2000 homology models were built using MODELLER v9.18 (San Francisco, CA, USA) [34]. PSIPRED v3.3 (London, UK) [35] secondary structure and TOPCONS v2.0 (Stockholm, Sweden) [36] transmembrane segment predictions were used to validate the models. The best GTR1_in_ model was selected as that with the lowest Molpdf energy value of MODELLER and the highest PROCHECK [37] and global ProQM [38] scores.

To model GTR1 in an outward-facing conformation (GTR1_out_), the NRT1.1 template structure was initially built in an alternate conformation employing the so-called repeat-swap homology modeling technique (see Figure 1) [39,40]. This protocol has been successfully used to obtain models of secondary active transporters in a given conformation using the X-ray structure of the transporter in the complementary conformation as a template. Four repeat units were identified in the NRT1.1 structure comprising residues 1–130 (repeat unit 1-A, RU1A), 131–245 (RU1B), 325–455 (RU2A) and 456–581 (RU2B). Structural superimposition of the repeat fragments were performed—i.e., RU1A/RU1B and RU2A/RU2B—using the TM-Align algorithm [41], which gives a sequence alignment between the repeats based on the pairs of residues that are close in space in the structural alignment. Superimposing the entirety of RU1A and RU1B yielded a TM score of 0.6, while between RU2A and RU2B, this value was 0.50. TM score measures the structural similarity between two protein fragments, ranging from 0 to 1, with 1 being structurally identical. An initial pairwise sequence alignment of the full-length protein was constructed by duplicating the sequence alignment obtained for RU1A/RU1B and RU2A/RU2B, as shown in Figure 1C. Thus, the RU1A of the protein was modeled based on, and is therefore aligned to, the RU1B, while the RU1B was modeled based on RU1A. The same strategy was used to model RU2A and RU2B. An initial swapped model of NRT1.1 was built using MODELLER v9.18 (San Francisco, CA, USA) [34]. Subsequently, due to the sequence and structural divergence of the repeats, it was necessary to remove gaps within secondary structure and several cycles of modeling refinement were carried out. A total of 2000 repeat-swapped NRT1.1_out_ models (in an outward-facing conformation) were generated. The best model was selected according to the PROCHECK and ProQM scores.

The best NRT1.1_out_ structure was then used to model GTR1_out_. The same sequence alignment used for modeling the GTR1_in_ (Figure S1) was used in this stage. A total of 2000 homology models were generated using MODELLER v9.18. The quality of the models was evaluated using PROCHECK and ProQM.

### 4.2. Molecular Modeling of GkPOT

To test if our ensemble docking workflow was actually accurate when predicting mayor binding sites into transporters, we selected the crystal structure of peptide transporter from *Geobacillus kaustophilus* (GkPOT) in complex with alafosfalin (PDB code: 4IKZ) as positive control [6]. This transporter was crystalized in the inward-facing conformation (GkPOT_in_), so we modeled the outward conformation (GkPOT_out_) using the same repeat-swap homology modeling protocol previously described for NTR1.1_out_. Four repeat units were defined, comprising the residues 21–117 (repeat unit 1 A, RU1A), 118–213 (RU1B), 286–384 (RU2A) and 385–494 (RU2B). A pairwise sequence alignment of the full-length protein was constructed by duplicating the sequence alignment obtained through the superimposition of RU1A/RU1B and RU2A/RU2B. Based on that alignment, an initial swapped model of the protein was built using MODELLER v9.218. A refined sequence alignment was then generated and used to build 2000 repeat-swapped GkPOT models. The best model was selected as that with the highest PROCHECK and ProQM scores (Figure S2).

### 4.3. Molecular Dynamics Simulations (MDs)

In order to equilibrate and relax all inward and outward transporters models, including GkPOT positive control, models were first prepared using the Protein Preparation Wizard included in Maestro. All the structures were optimized at pH 4.5 ± 2.0 with Epik [42] and PROPKA [43] because GTRs actively transport glucosinolates at low pH [14,18,19]. Structures were minimized converging heavy atoms to RMSD = 0.3 Å, and then embedded into a pre-equilibrated phosphatidyl oleoylphosphatidylcholine (POPC) bilayer in a periodic boundary condition box with pre-equilibrated TIP3 water molecules. Na^+^ or Cl^−^ ions were added to neutralize the systems, and then the ion concentration was set to 0.15 M NaCl. Prior to equilibrium simulations, the systems were relaxed using the default Desmond’s relaxation protocol. Then, the systems were equilibrated for 5 ns in an NPT ensemble at 310 K with the application of a restraint spring constant of 20 kcal·mol^−1^·Å^−2^ to the protein backbone atoms, followed with another 5 ns but reducing the restraint spring constant to 10 kcal·mol^−1^·Å^−2^. After a proper system equilibration, a 200 ns production MDs in NPT ensemble was performed applying a restraint spring constant to 1 kcal·mol^−1^·Å^−2^ to the secondary structure of each transporter. In both equilibrium and production MDs, temperature and pressure were kept constant at 310 K and 1.01325 bar respectively by coupling to a Nose–Hoover Chain thermostat [44] and Martyna–Tobias–Klein barostat [45] with an integration time step of 2 fs. MDs were performed with Desmond [46] and the OPLS3 force field [47]. The simulations were analyzed with Desmond, KNIME, Schrödinger, and *in-house* scripts. Visualization was carried out with VMD [48] and Pymol [49].

After relaxation, from each system, 100 frames were extracted from the last 100 ns of the simulation (every 1 ns). The transporter structures were extracted and aligned using the *trj2mae.py* script included in Schrödinger suite. At the end, 400 structures in total were obtained with this protocol: 100 structures per model (four models: GTR1_in_, GTR1_out_, GkPOT_in_, and GkPOT_out_—Table 1).

### 4.4. Ensemble Docking of 4MTB and I3M into GTR1 Transporter

To find the best glucosinolate binding mode interacting with GTR1, and considering the flexibility of the transporter, we performed an ensemble docking in the structures collected every 1 ns from the last 100 ns of simulation for each system using the software Glide [50] and the Standard Precision (SP) function, obtaining 10 poses per docking simulation. Glucosinolates 4MTB and I3M were docked into GTR1 in both inward and outward conformations, and alafosfalin was docked into GkPOT in both inward and outward conformations. Alafosfalin results were compared with the crystallographic structure PDB code 4IKZ as positive control of our ensemble docking pipeline. Glucosinolates were downloaded from the Plant Secondary Compound database [51] and alafosfalin was obtained from the crystallographic structure [6]. All ligands were processing using LigPrep [52]. Incorporation of conformational rearrangements of the transporter binding pocket into predictions of the substrate binding mode was critical for improving docking results [53,54]. The substrate-binding site was defined by the residues lining the pore in both inward- and outward-facing conformations; in this way, we ensured that all residues that potentially interact with substrates when the substrate is translocated from outside to inside the cell were included in the grid box. The molecular docking simulations were carried out with the outer box edge of the grid setting as (32_x_ × 32_y_ × 42_z_) Å^3^. At the end, the following systems were studied by ensemble docking: GTR1_in_-4MTB, GTR1_out_-4MTB, GTR1_in_-I3M, and GTR1_out_-I3M. As positive control, we used the systems GkPOT_in_-alafosfalin and GkPOT_out_-alafosfalin.

### 4.5. Clustering and Docking Postprocessing

To process and organize the docking solutions per system, we used the conformer cluster script included in Maestro. This script builds a matrix [55] using a measure of pairwise distance between conformations. This measure was the root mean square displacement (RMSD) between pairs of corresponding atoms following optimal rigid-body superposition [56]. The atomic RMSD was calculated considering the heavy atoms from each substrate, and the linkage average method was used to cluster the docking poses. Significant conformational clusters (SC) were identified considering the total number of clusters obtained per system using the following equation:

(1) $$SC=\bar{x}+2\delta ,$$

where $\bar{x}$ is the average cluster population per system, and δ is the standard deviation [57,58]. SC were considered for further analysis.

Interactions between each substrate conformer and its corresponding transporter conformation for all significant clusters were profiled using the *poseviewer_interactions.py* script included in Schrödinger suite. The following contacts were studied for each complex: hydrogen bonds (H-bonds), halogen bonds, salt bridges (ionic), Pi–cation, Pi–Pi, hydrophobic, water-mediated hydrogen bonds, aromatic hydrogen bonds, and metal contacts. Both glucosinolates were broken down into three different moieties to profile the interactions: sugar, sulfate, and sidechain. Sugar and sulfate moieties form part of the common ligand’s backbone, and the sidechain is different in both glucosinolates, as 4MTB has an aliphatic sidechain (derived from methionine) and I3M has an indole group (derived from tryptophan) (Figure 5). With the *poseviewer_interactions.py* script, we tracked down—for all the conformers on each significant cluster—which ligand atom (from sugar, sulfate, or sidechain moieties) interacts with a given GTR1 residue, and then calculated the frequency of the interaction per cluster ($FI_{PC}$). Later, the interactions were weighted by each cluster using the significant cluster population (SCP—Table S1), as described in Equation (2):

(2) $$FI=\frac{FI_{PC}\times SCP\;}{TPSC}$$

where FI is the frequency interaction and TPSC is the total population of all significant clusters per system (Table S1). Finally, all FI per residue were summed up to weigh how many interactions a specific residue had with the conformers of the significant clusters. This post-docking analysis was fully automated by using an *in-house* KNIME workflow [59] which is now available free of charge to the entire community at https://bit.ly/3lc1yqa.

### 4.6. Cloning of Mutants for Xenopus Oocyte Expression and cRNA Generation

All GTR1 mutant versions were cloned into *Xenopus* expression vectors, either pNB1u or pNB1uYFP [60]. All mutants were created by USER fusion [61]. Briefly, the CDS of GTR1 was amplified in two fragments via PCR. Fragment 1 was amplified using the standard USER forward primer and a reverse primer annealing close to the mutation site. Fragment 2 was amplified using a forward primer annealing close to the mutation site (overlapping with part of the reverse primer from fragment 1) and a reverse primer in the end of the sequence. Either one or both of the primers flanking the mutation site contained the point mutation. All PCR fragments had a uracil incorporated at both ends through the primers. Primers used for PCR amplification are listed in primer Table S2.

Linearized DNA template for RNA synthesis were generated by PCR amplification using forward primer (5′-AATTAACCCTCACTAAAGGGTTGTAATACGACTCACTATAGGG-3′) and reverse primer (5′-TTTTTTTTTTTTTTTTTTTTTTTTTTTTTATACTCAAGCTAGCCTCGAG-3) [62]. PCR products were purified using E.Z.N.A Cycle Pure Kit (Omega Bio-tek) using the manufacturer’s instructions. The PCR products were in vitro transcribed using the mMessage mMachine T7 transcription kit (InVitrogen) using the manufacturer’s instructions; cRNA concentrations were adjusted to 500 ng/μL.

### 4.7. Xenopus Oocytes Transport Assay

Defolliculated *Xenopus laevis* oocytes were purchased from Ecocyte Biosciences. The oocytes were injected with 50.6 nL cRNA using a Drummond Nanoject II. Before use in assays, the oocytes were incubated for 3 days at 16 °C in HEPES-based kulori (90 mM NaCl, 1 mM KCl, 1 mM MgCl_2_, 1 mM CaCl_2_, 5 mM HEPES, pH 7.4). The expressing oocytes were preincubated in MES-based kulori (90 mM NaCl, 1 mM KCl, 1 mM MgCl_2_, 1 mM CaCl_2_, 5 mM MES, pH 5) for 2 min, before being transferred to glucosinolate-containing MES-based kulori for 1 h. After incubation, oocytes were washed three times in HEPES-based kulori followed by one wash in MilliQ water. Single oocytes were homogenized in 50% methanol containing internal standard (2-propenyl (sinigrin), 1250 nM) and stored for at least 30 min at −20 °C. Following 10 min centrifugation at 4 °C at minimum 19.000 g, the supernatant was diluted with MilliQ water to a final methanol concentration of 20%. The supernatant was filtered through a 0.22 µm filter plate (Merck Millipore, MSGVN2250) before being run on LCMS.

### 4.8. Glucosinolate Analysis by LC-MS/MS

The diluted oocyte samples were subjected to analysis by liquid chromatography coupled to tandem mass spectrometry. The method was modified from Crocoll et al. (2016), and parameters were adjusted and optimized to match the LC-MS/MS system in use [63]. Briefly, chromatography was performed on a 1290 Infinity II UHPLC system (Agilent Technologies). Separation was achieved on a Kinetex XB-C18 column (100 × 2.1 mm, 1.7 µm, 100 Å, Phenomenex, Torrance, CA, USA). Formic acid (0.05%, *v/v*) in water and acetonitrile (supplied with 0.05% formic acid, *v/v*) were employed as mobile phases A and B, respectively. The elution profile for glucosinolates was: 0–0.2 min, 2% B; 0.2–3.5 min, 2–45% B; 3.5–4.2 min, 45–100% B; 4.2–4.9 min, 100% B; 4.9–5.0 min, 100–2% B; 5.0–6.0 min, 2% B. The mobile phase flow rate was 400 µL/min. The column temperature was maintained at 40 °C. The liquid chromatography was coupled to an Ultivo Triplequadrupole mass spectrometer (Agilent Technologies) equipped with a Jetstream electrospray ion source (ESI) operated in negative ion mode. The instrument parameters were optimized by infusion experiments with pure standards. The ion spray voltage was set to 4500 V. Dry gas temperature was set to 325 °C and dry gas flow to 13 L/min. Sheath gas temperature was set to 400 °C and sheath gas flow to 12 L/min. Nebulizing gas was set to 55 psi. Nitrogen was used as dry gas, nebulizing gas, and collision gas. Multiple reaction monitoring (MRM) was used to monito ion → fragment ion transitions. MRM transitions were determined by direct infusion experiments of reference standards. Detailed values for mass transitions can be found in Supplementary Table S3. Both Q1 and Q3 quadrupoles were maintained at unit resolution. Mass Hunter Quantitation Analysis for QQQ software (Version 10, Agilent Technologies) was used for data processing. Linearity in ionization efficiency was verified by analyzing dilution series that were also used for quantification of glucosinolates in the samples.

## Figures and Tables

**Table 1:** Transporters used and modeled in this study.

**Crystallographic Structures (Inward-Facing Conformation)**			
NTR1.1_in_ (PDB code: 4OH3)		GkPOT_in_ (PDB code: 4IKZ)	
**Model in an inward-facing conformation**			
GTR1_in_			
**Models in an outward-facing conformation**			
NTR1.1_out_	GkPOT_out_	GTR1_out_	

**Table 2:** Population of alafosfalin significant clusters.

Cluster Nº	GkPOT_in_	GkPOT_out_
1	100	142
2	96	142
3	95	91
4	87	86

**Table 3:** Summary of massive docking solutions clustering process.

	GTR1_in_-4MTB	GTR1_in_-I3M	GTR1_out_-4MTB	GTR1_out_-I3M
	98	186	510	184
	81	173		96
	73			88
	70			
	53			
	50			
	49			
# Significant cluster	7	2	1	3
Total population	986	965	979	993
Total population Significant clusters	474	359	510	368
% conformers in significant clusters	48.07%	37.20%	52.09%	37.06%

**Table 4:** Key interacting residues of GTR1 identified with ensemble docking of approximation along with homologous residues from POT crystallographic structures.

GTR1	GkPOT (PDB: 4IKZ) [6]	PepT_St_ (PDB: 4D2C) [20]	PepT_St_ (PDB: 4D2D) [20]	Function [14]
E75	**E32**	E22	E22	ExxE[K/R] motif [6,14,15]
E78 *	**E35**	E25	E25	ExxE[K/R] motif [6]
K79 *	**R36**	R26 *	**R26**	ExxE[K/R] motif [6,15]
I82	Y39	Y29	**Y29**	Peptide specificity [21]
**I83**	Y40 *	Y30 *	**Y30**	Peptide specificity [6]
L86	R43 *	R33	**R33**	Peptide specificity [6]
N116 *	Y78 *	**Y68**	**Y68**	Peptide binding [6]
T119 *	G81	G71	G71	------
**L200**	S140	S130	S130	------
R196 *	**K136**	K126 *	K126	ExxE[K/R]-interactor [6]
F222	F158	F148	F148	------
**F226**	Y162	V152	V152	------
**F229**	I165	**I155**	I155	------
**T230**	**N166**	**N156**	N156	Peptide binding [6]
**Q233**	A169	**A159**	A159	------
**Y382**	W306	**W296**	**W296**	------
**I385**	Q309	**E299**	**E299**	Peptide binding/protonation [2,3]
T386	E310Q *	E300	**E300**	------
**M389**	S313	S303	S303 *	------
**L419**	N342 *	N328 *	**N328**	Peptide binding [6]
**M420**	P343	**P329**	P329	------
**E513**	**E413**	**E400**	E400 *	Proton translocation [6]
**A517**	S417	S404	S404	------
**F540**	W440	**W427**	W427	------
Y541	F441	**F428**	F428	------
A544	N444	S431	**S431**	------
S547 *	A447	G434	G434	------

**GTR1-bold:** Residues identified through ensemble docking approximation. **Gray:** Residues identified in POTs crystallographic structures. * Indicates H-bond interactions. The residues in the rows are homologous in all structures according to the multiple sequence alignment of sequences reported by [14].

## Data Availability

The computational pipeline, as well as KNIME workflow to profile interactions, is available at https://bit.ly/3lc1yqa.

## Supplementary Materials

The following are available online at https://www.mdpi.com/article/10.3390/ijms23031595/s1.
