# Supplementary material for: Structural Insights into the Substrate Transport Mechanisms in GTR Transporters through Ensemble Docking
Source: Int J Mol Sci. 2022 Jan 29;23(3):1595. doi: 10.3390/ijms23031595 (PMC8836200; doi:10.3390/ijms23031595)
Supplement: Supplementary file 1 [file ijms-23-01595-s001.zip › ijms-1565123-supplementary.pdf]

Structural insights into the substrate transport mechanisms in GTR transporters through ensemble docking.

Supplementary data

Supplementary Figures

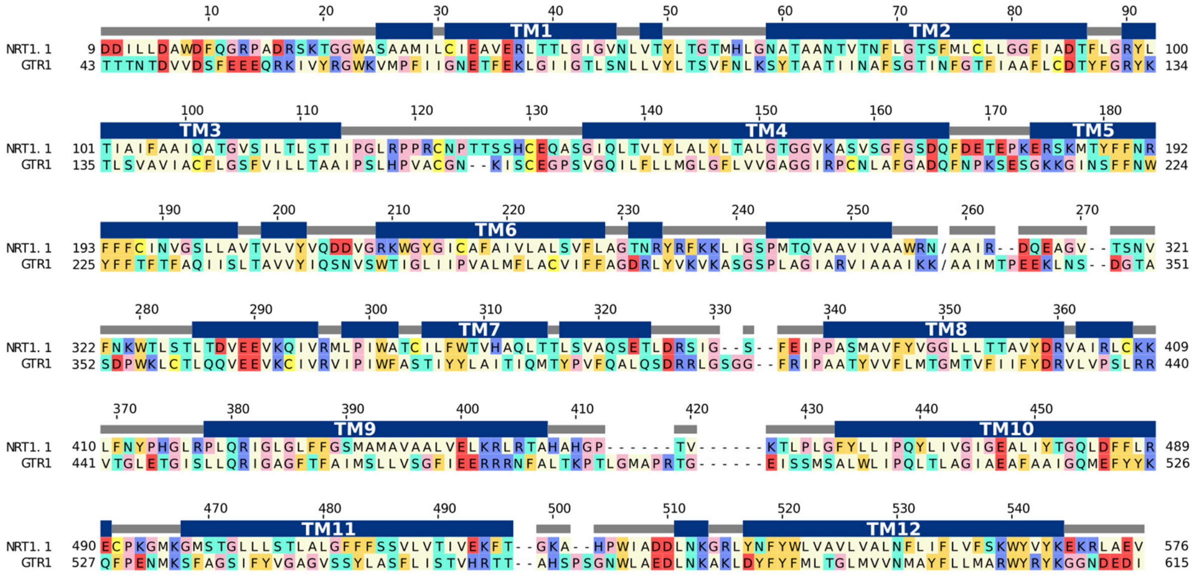

Figure S1: Sequence alignment between NRT1.1 and GTR1 used for modeling. The alignment is colored according to the chemical properties of the residues: light yellow, aliphatic (A, I, L, M, and V); cyan, polar uncharged (N, Q, S, and T); orange, aromatic (F, W, and Y); red, acidic (D and E); purple, basic (K, R, and H); pink, exceptional (G and P), and yellow (C). The secondary structure (helix) assignment for the NRT1.1 crystal structure was obtained with DSSP and is indicated by dark blue rectangles. The sequence identity between NRT1.1 and GTR1 is 30.3%.

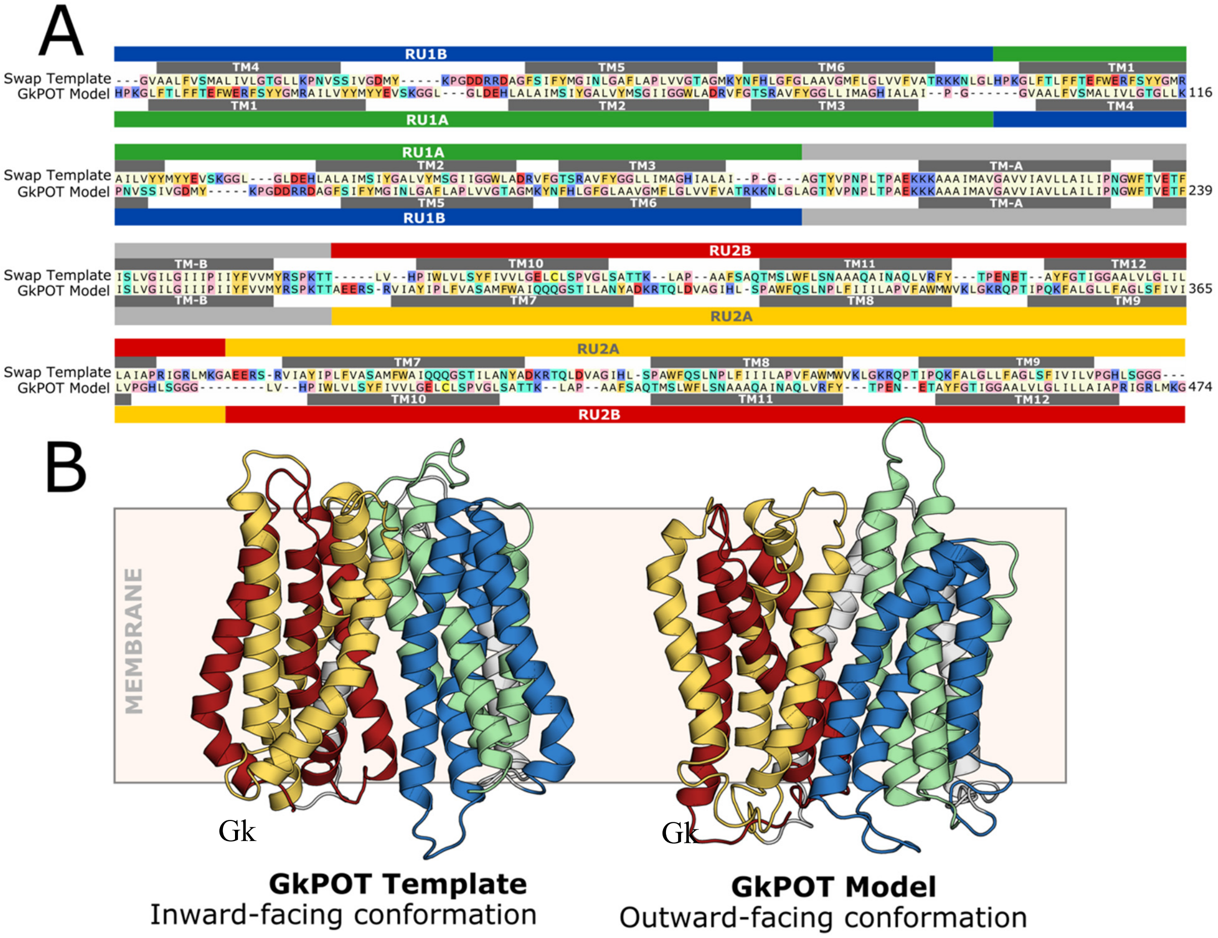

Figure S2: Repeat-swap modeling of GkPOT in an outward-facing conformation. (A) Refined sequence alignment between the GkPOT and swapped-template sequences. The sequence alignment is colored according to the chemical properties of the residues. The secondary structure (helix) assignment is indicated by dark grey rectangles and labeled by TM segments. The sequence range of each repeat is displayed with colored bars above and under the template and target sequences. The region comprising RU1A, RU1B, RU2A and RU2B are colored in green, blue, yellow, and red, respectively. (B) The GkPOT structure in an inward-facing conformation (left) is compared with the repeat-swapped model in an outward-facing conformation (right). Structures are viewed along the plane of the membrane, with the extracellular side at the top.

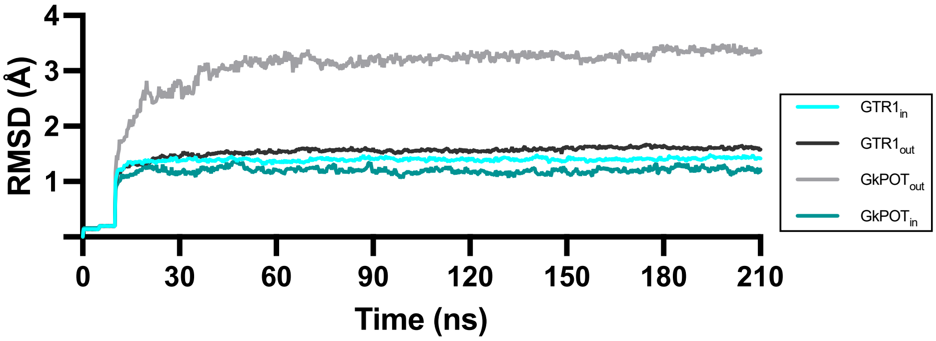

Figure S3. RMSD for GTR1 and GkPOT atoms during the MDs. A restraint spring constant of 20, 10 and 1 kcal × mol^{-1} × $Å^{-2}$ was applied between 0-5 ns, 5-10 ns, and 10-210 ns, respectively.

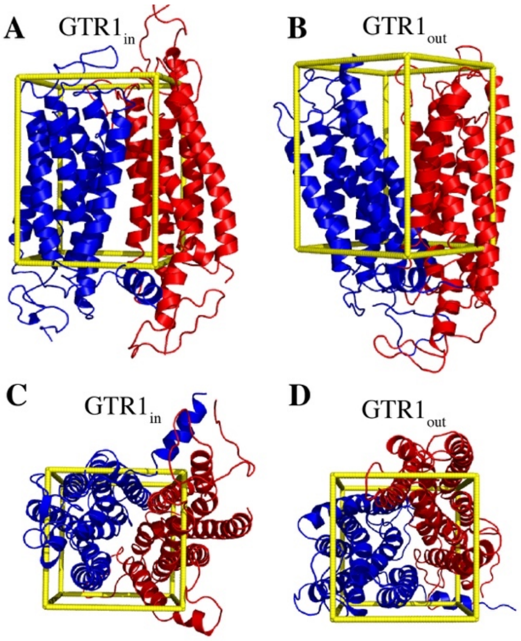

Figure S4. Grid box used in ensemble docking. $GTR1_{in}$ (A, C) and $GTR1_{out}$ (B-D) models with grid boxes represented as yellow lines. (A-B) Side-view representation. (C) Bottom-view representation. (D) Up-view representation. Residues lining the central cavity are always contained by the grid box. The outer box edge of all grid boxes used is (32_x × 32_y × 42_z) $Å^3$.

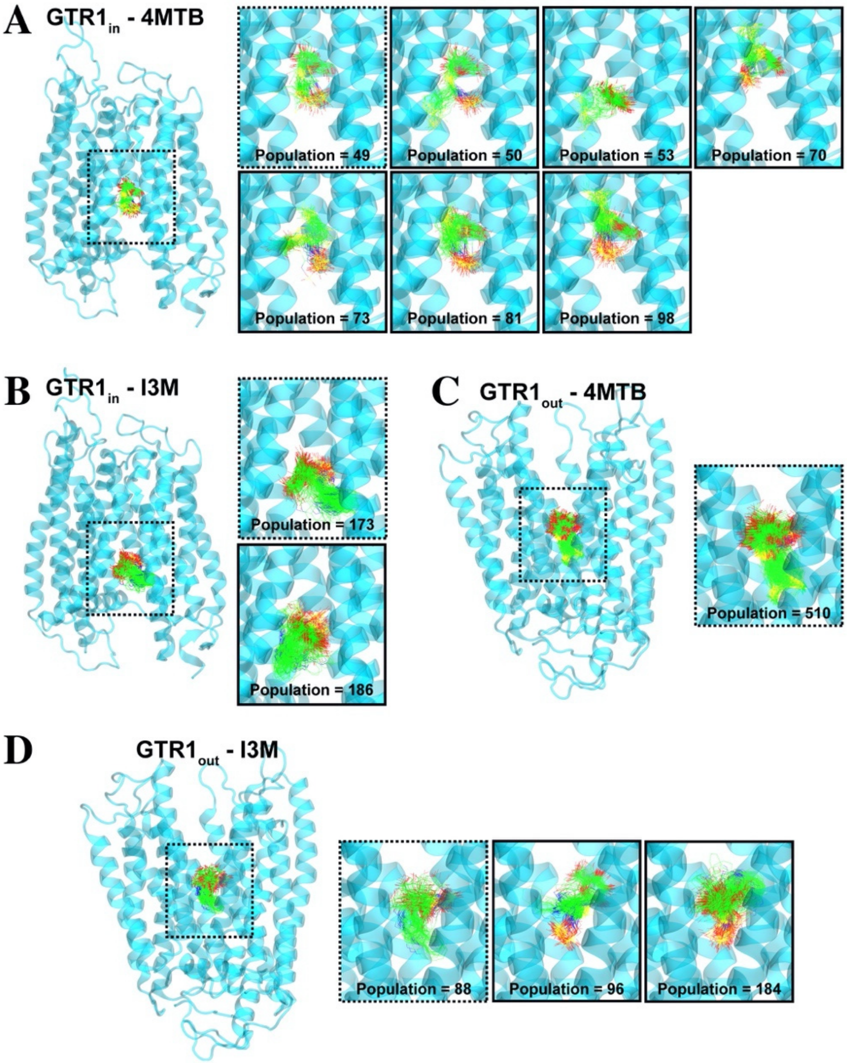

Figure S5. Ensemble docking of glucosinolates into GTR1. Significant clusters of 4MTB (A, C) and I3M (B, D) conformers from ensemble docking into GTR1 in both inward and outward conformations.

Supplementary Tables

Table S1. Clusters population obtained from ensemble docking of glucosinolates into GTR1.

$GTR1_{in}$ - 4MTB	$GTR1_{out}$ - 4MTB	$GTR1_{in}$ - I3M	$GTR1_{out}$ - I3M
98	510	186	184
81	81	173	96
73	42	61	88
70	34	54	57
53	27	53	54
50	21	42	47
49	19	36	30
40	17	34	29
35	17	33	21
34	16	32	20
31	15	25	20
27	14	21	19
20	13	16	17
17	8	16	17
15	8	15	14
14	7	14	13
13	7	13	13
12	6	13	12
11	6	13	11
10	6	11	10
9	6	11	10
9	5	11	10
8	5	7	8
8	5	7	8
7	5	7	8
7	5	6	7
7	4	5	6
7	4	5	6
6	4	5	6
6	3	3	6
6	3	3	5
6	3	3	5
6	3	3	5
6	3	3	5
6	3	2	5
5	3	2	5
5	3	2	5

4	2	2	4
4	2	2	4
4	2	2	4
4	2	1	4
4	2	1	4
4	2	1	4
4	2	1	3
4	2	1	3
3	2	1	3
3	2	1	3
3	2	1	3
3	2	1	3
3	1	1	3
3	1	1	3
3	1	1	3
3	1	1	2
3	1		2
3	1		2
3	1		2
3	1		2
3	1		2
3	1		2
3	1		2
2	1		2
2	1		2
2	1		2
2			2
2			2
2			2
2			2
2			2
2			2
1			1
1			1
1			1
1			1
1			1
1			1
1			1
1			1

	1			1
	1			1
	1			1
	1			1
	1			1
	1			1
	1			1
	1			1
	1			1
	1			1
	1			1
	1			1
	1			1
	1			1
	1			1
	1			1
				1
				1
**Total pop**	986	979	965	993
**Average pop**	10.60	15.54	18.21	10.45
**SD pop**	18.67	64.51	35.61	24.11
**Sig Cluster threshold**	47.95	144.56	89.43	58.68
**Number of Sig Cluster**	7	1	2	3
**Total pop Sig Cluster**	474	510	359	368
**% conformers in significant clusters**	48.07%	52.09%	37.20%	37.06%

pop: Population; **SD:** Standrad deviation; **Sig cluster:** Significant clusters

Table S2. Primers used in this study.

No	GTR1 version	vector	Fragment	Primer direction	Primer sequence
1	GTR1_R196K	pNB1u	1	Forward	GGCTTAAUATGAAGAGCAGAGTCATTCTTAACC
				Reverse	ACGGTgTGAUACCACCAGCG
			2	Forward	ATCAaACCGUGTAATTTAGCGTTTGG
				Reverse	GGTTTAAUTCAGACAGAGTTCTTGTCTTG
2	GTR1_R196Q	pNB1u	1	Forward	GGCTTAAUATGAAGAGCAGAGTCATTCTTAACC
				Reverse	ACGGTtgGAUACCACCAGCG
			2	Forward	ATCcaACCGUGTAATTTAGCGTTTG
				Reverse	GGTTTAAUTCAGACAGAGTTCTTGTCTTG
3	GTR1_R196A	pNB1uYFP	1	Forward	GGCTTAAUATGAAGAGCAGAGTCATTCTTAACC
				Reverse	ACGGTgcGAUACCACCAG
			2	Forward	ATCgcACCGUGTAATTTAGCG
				Reverse	GGTTTAAUCCGACAGAGTTCTTGTCTTGTAG
4	GTR1_E513D	pNB1uYFP	1	Forward	GGCTTAAUATGAAGAGCAGAGTCATTCTTAACC
				Reverse	AGCaTCTGCTAUGCCTGC
			2	Forward	ATAGCAGAtGCUTTTGCTGCC
				Reverse	GGTTTAAUCCGACAGAGTTCTTGTCTTGTAG
5	GTR1_E513Q	pNB1uYFP	1	Forward	GGCTTAAUATGAAGAGCAGAGTCATTCTTAACC
				Reverse	AGCTTgTGCTAUGCCTGC
			2	Forward	ATAGCAcAAGCUTTTGCTGCC
				Reverse	GGTTTAAUCCGACAGAGTTCTTGTCTTGTAG
6	GTR1_E513A	pNB1uYFP	1	Forward	GGCTTAAUATGAAGAGCAGAGTCATTCTTAACC
				Reverse	AGCTgCTGCTAUGCCTGC
			2	Forward	ATAGCAGcAGCUTTTGCTGCC
				Reverse	GGTTTAAUCCGACAGAGTTCTTGTCTTGTAG

Red marks uracil in the primers. Underlined lower case marks the mutation.

Table S3: MRM transitions for glucosinolates quantified by LC-MS/MS.

Analyte	Retention Time [min]	Q1 [*m/z*]	Q3 [*m/z*]	Fragmentor [V]	CE [V]
2-propenyl (sinigrin)	1.72	358.0	97.0^{Qt}	98	20
$[M-H]^-$		358.0	259.0	98	16
Internal standard (IS)		358.0	75.0	98	36
4mtb	2.56	420.0	97.0^{Qt}	107	24
[M-H]-		420.0	259.0	107	20
		420.0	75.0	107	40
I3M	2.69	447.0	97.0^{Qt}	112	24
[m-H]-		447.0	259.0	112	24
		447.0	205.0	112	20

Qt = quantifier ion, additional transitions were used for identification. Q = quadrupole. CE = collision energy.
